# Efficacy and safety of ibuprofen gargle for postoperative pain after mandibular third molar extraction: A phase II, placebo‐controlled, double‐blind, randomized crossover trial

**DOI:** 10.1002/cre2.821

**Published:** 2023-11-29

**Authors:** Takeshi Ioroi, Yasumasa Kakei, Takahiro Ito, Tatsuya Shirai, Yutaro Okazaki, Takumi Hasegawa, Masaya Akashi, Ikuko Yano

**Affiliations:** ^1^ Department of Pharmacy Kobe University Hospital Hyogo Japan; ^2^ Department of Oral and Maxillofacial Surgery Kobe University Graduate School of Medicine Hyogo Japan

**Keywords:** ibuprofen, molar, mouthwashes, postoperative pain, third

## Abstract

**Objective:**

This study was designed to evaluate the postoperative efficacy and safety of using an ibuprofen gargle as a pain management strategy for patients who have undergone mandibular third molar extraction. We also ensured that the quality of treatment was not compromised throughout the study.

**Material and Methods:**

Patients were randomized in a 1:1 ratio into two groups: the ibuprofen–placebo (IP) group and the placebo–ibuprofen (PI) group. On postoperative Day (POD) 1, the IP group initiated ibuprofen administration, while the PI group started taking placebo. On POD 2, the IP group switched to using placebo, whereas the PI group switched to ibuprofen. From PODs 3–5, both groups were prescribed ibuprofen gargle. The primary endpoint was within‐subject visual analog scale (VAS) score before and 5 min after the first use of the ibuprofen or placebo gargle on PODs 1 and 2 (ΔVAS_5_ibuprofen_ − ΔVAS_5_placebo_). The incidence and severity of adverse events were assessed using the Common Terminology Criteria for Adverse Events version 5.0 and a subjective rating scale.

**Results:**

This study enrolled 40 patients. The within‐subject VAS_5_ of the IP and PI groups were 1.25 ± 12.0 and −5.26 ± 8.93 mm, respectively. The treatment effect of ibuprofen gargle was −2.01 ± 10.62 mm (*p* = .246). None of the patients in each group presented with serious adverse events or clinically significant complications (including dry sockets) after extraction. Transient adverse events, such as throat tingling and oral discomfort (grade 1), were observed in each group.

**Conclusion:**

Ibuprofen gargle was safe but did not provide significant pain relief when used after mandibular third molar extraction.

## 1 INTRODUCTION

Extraction of mandibular third molars, which may involve bone removal and crown sectioning, is a common oral surgical procedure associated with moderate to severe postoperative complications (Fraga et al., 2020; O'Hare et al., 2019). Although not all mandibular third molar extractions involve bone removal and crown sectioning, the potential risks associated with these invasive techniques should be carefully considered. The most common complications of this type of surgery include pain, swelling, difficulty in opening the mouth and swallowing (Cicciù & Sortino, 2011; O'Hare et al., 2019), and challenges associated with oral consumption.

Tooth extraction is usually painful; therefore, several patients are reluctant to undergo therapy (Fraga et al., 2020). While recent studies have explored the potential of platelet‐rich fibrin (PRF) and advanced platelet‐rich fibrin (A‐PRF+) to manage postoperative pain after third molar extractions (He et al., 2017; Vitenson et al., 2022), the predominant approach remains the use of analgesics. Among these, nonsteroidal anti‐inflammatory drugs (NSAIDs) and acetaminophen stand out as the most effective (Hersh et al., 2020; Pergolizzi et al., 2020). Acidic NSAIDs are typically prescribed for mild to normal pain, which includes pain associated with mandibular extractions (Hargreaves & Abbott, 2005; Miroshnychenko et al., 2023; Orozco‐Solís et al., 2016). Acetaminophen presents itself as a viable therapeutic option for patients who are experiencing gastrointestinal ulcers or asthma associated with the use of aspirin (Kacso & Terézhalmy, 1994; Pergolizzi et al., 2020). Previous studies have assessed whether NSAIDs, such as celecoxib, valdecoxib, ibuprofen, flurbiprofen, lornoxicam, etoricoxib, and opioid‐containing drugs (such as oxycodone) (Benetello et al., 2007; Demirbas et al., 2019; Franco‐de la Torre et al., 2021; Isola, Alibrandi, et al., 2019; Isola, Matarese, Ramaglia, Cicciù, et al., 2019; Pruskowski et al., 2019; Rajanna & B R, 2021; Xie et al., 2020), are the most effective analgesics in patients who underwent mandibular third molar extractions. Notably, flurbiprofen, lornoxicam, and a phytotherapeutic compound consisting of baicalin, bromelain, and escin have been identified as efficacious in managing pain following third molar surgery (Isola, Alibrandi, et al., 2019; Isola, Matarese, Ramaglia, Iorio‐Siciliano, et al., 2019). Additionally, the oral administration of paracetamol (acetaminophen) has demonstrated both safety and efficacy in providing pain relief after undergoing embedded mandibular third molar extraction (Dodson, 2007, p. 1; Weil et al., 2007; Weyant, 2009). In the treatment of postoperative pain, oral ibuprofen was found to be more effective than oral paracetamol (Bailey et al., 2013; Moore et al., 2015).

Analgesics should be prescribed in the postoperative period with consideration of side effects. Opioids have been linked to respiratory depression as well as nausea, vomiting, and constipation (Bailey et al., 2013). Meanwhile, NSAIDs have been linked to gastrointestinal disorders and potential systemic dysfunction in platelets, kidneys, and the liver (Gupta & Bah, 2016). A cluster‐randomized controlled trial on interventions to decrease opioid prescriptions in favor of nonopioids was recently published (Gryczynski et al., 2023). Although all study arms showed a significant decrease in opioid prescriptions, providing clinical decision support resources to dental providers did not result in greater reductions in opioid prescriptions after tooth extractions (Gryczynski et al., 2023). Furthermore, for postoperative pain relief, topical analgesics, such as zinc lozenge, chlorhexidine gel, and diclofenac hydroxypropyl‐β‐cyclodextrin, have been suggested as feasible alternatives to traditional oral and injectable pain medications (Gorecki et al., 2018; Lopez‐Lopez et al., 2015; Rajanna & B R, 2021). These topical analgesics are recognized for their ability to deliver effective pain relief while minimizing the occurrence of adverse drug reactions (Mishra et al., 2017).

During the 1960s, ibuprofen was developed as an inhibitor of prostaglandin synthesis to reduce symptoms of fever, pain, and inflammation (Rainsford, 2009). Although other effects have been reported (Neychev et al., 2020), ibuprofen is known to inhibit both cyclooxygenase (COX)‐1 and COX‐2. Hence, it may cause gastrointestinal and renal dysfunction if administered systemically. Multiple reviews and meta‐analyses have reported that ibuprofen exhibits a comparatively low level of toxicity among NSAIDs and that it is effective in treating both children and adults (Barbagallo & Sacerdote, 2019; de Martino et al., 2017; Varrassi et al., 2020). We conceived that oral gargling with a solution of dissolved ibuprofen (0.6% or 1%) applied directly to the affected area of oral mucositis could provide pain relief. Because of the loss of the permeability barrier in oral mucosa (Patel et al., 2011), we assumed that drug diffusion into tissues is faster than that in intact mucosal areas. According a previous study, ibuprofen gargle could relieve chemotherapy‐ or chemoradiotherapy‐associated oral mucositis (Ioroi et al., 2020).

The absence of keratinized mucosa enhances the rapid absorption of medications, which in turn augments the efficacy of topical medications. This is even true for wounds caused by tooth extraction, which have a distinct pathogenesis from mucositis (Isola, Matarese, Ramaglia, Iorio‐Siciliano, et al., 2019; Pergolizzi et al., 2020; Xie et al., 2020). Topical NSAIDs have been reported to be effective after tooth extraction (Dionne et al., 1999; Moore et al., 1992). However, the efficacy and safety of ibuprofen‐containing gargles, when used as a rinse, remain uncharted territory.

Given that topical NSAIDs may offer targeted pain relief without the systemic side effects observed with oral administration, it becomes imperative to comprehensively assess the potential benefits and risks of this administration mode following tooth extraction.

In an effort to bridge the prevailing knowledge gap, this research focuses on evaluating the efficacy and safety of ibuprofen‐containing gargles administered via gargling. Specifically, the study aims to assess the impact of ibuprofen gargle on postoperative pain in patients who have undergone mandibular third molar extraction, ensuring that treatment quality is not compromised.

## 2 MATERIALS AND METHODS

### 2.1 Consent to participate and ethical approval

Before conducting any study procedure, written informed consent was obtained from all patients, indicating their full comprehension of the details of the procedure. A qualified and experienced individual distributed the informed consent forms. The Clinical Research Ethics Committee approved the study protocol of Kobe University (reference number: C200024, date of approval: March 23, 2021). The study was performed in accordance with the principles of the Declaration of Helsinki and Clinical Trial Acts in Japan. The trial was registered in the Japan Registry of Clinical Trials (trial registration: jRCTs051210022, date of registration: May 10, 2021).

### 2.2 Study design and population

This was a single‐center, placebo‐controlled, double‐blind, randomized crossover study. It was performed at Kobe University Hospital. This study was designed and conducted according to the CONSORT statement guidelines (Dwan et al., 2019). Patients undergoing third molar extraction were selected based on the defined inclusion and exclusion criteria, with the first patient enrolled on June 7, 2021, as described in the following section.

### 2.3 Inclusion and exclusion criteria

#### 2.3.1 Inclusion criteria

1. Patients who planned to undergo lower third molar extraction and who were expected to register on the extraction day
2. Patients aged ≥ 20 years
3. Patients who were classified as Pell–Gregory (space) Class I or II
4. Patients who were classified as Pell–Gregory (depth) Class A or B
5. Patients who did not require general anesthesia for tooth extraction
6. Patients whose teeth were extracted by one of two experienced oral surgeons (Y. K. with 12 years and TS with 5 years of postgraduate training) using standard extraction methods.

#### 2.3.2 Exclusion criteria

1. Patients with peptic ulcers
2. Patients with concurrent severe or uncontrollable conditions
3. Patients with a history of hypersensitivity to any component of the ibuprofen gargle
4. Patients with impaired cardiac function or clinically significant heart disease
5. Patients with aspirin‐induced asthma
6. Patients with chronic pain who administered analgesic drugs at least once weekly
7. Patients with dementia, psychiatric symptoms, drug addiction, or alcoholism
8. Pregnant or lactating women
9. Patients who were deemed by the Investigator to be unable to comply with the study protocol.

### 2.4 Surgical procedure and prohibited interventions

According to a standardized protocol, surgeries were performed by experienced oral surgeons (Y. K. and T. S.) with at least 3 years of postgraduate experience. Further information on the surgical procedures is provided in our previous report (Kakei et al., 2022).

All patients included in the study underwent surgery performed by an oral surgeon with a minimum of 3 years of postgraduate experience, following a standardized protocol. Before administering the blockage of the inferior alveolar nerves, the oral surgeon should first use a povidone‐iodine solution to disinfect and sterilize the surface of the affected area in the patient's mouth. Following the disinfection, a 2% lidocaine + 1:80,000 epinephrine injection is administered. Subsequently, a mucoperiosteal flap was elevated using the Partial Newmann flap method, a standard incision technique in our practice(K et al., 2018). Bone removal and tooth sectioning were performed with a low‐speed handpiece under sterile irrigation if deemed necessary. Following tooth extraction, the socket was irrigated with 10–20 mL of saline, and the flap was closed using a few absorbable sutures (3−0 Vicryl; Ethicon Inc). The duration of the entire surgical procedure, from incision to the placement of the last suture, was recorded in minutes. Additional local analgesia should be administered and documented during surgery if required. Following surgery, patients should be given loxoprofen tablets (60 mg; 180 mg daily) for pain relief. Antibiotics should be prescribed and administered until postoperative Day (POD) 2.

### 2.5 Ibuprofen gargle

The ibuprofen gargle was manufactured at the Department of Pharmacy, Kobe University Hospital. The gargle (100 mL) contained ibuprofen (600 mg, 0.6%; Tokyo Chemical Industry), sodium hydroxide (Fujifilm Wako), sodium hydrogen carbonate (Fujifilm Wako), hydrochloric acid (for pH regulation; Fujifilm Wako), glycerin (Fujifilm Wako), and flavor (Marugo). The placebo gargle formulation was designed to be similar to the ibuprofen gargle in terms of taste and appearance, but without the inclusion of ibuprofen.

### 2.6 Treatment protocol

On POD 1, the ibuprofen–placebo (IP) group was instructed to use ibuprofen gargle, whereas the placebo–ibuprofen (PI) group was instructed to use placebo gargle. On POD 2, the IP group switched to placebo gargle, whereas the PI group switched to ibuprofen gargle. From PODs 3–5, both groups were prescribed ibuprofen gargle at least once daily. Given that several studies have indicated the peak of pain after third molar extraction to occur around 1−3 days postoperatively (Avellaneda‐Gimeno et al., 2017; Colorado‐Bonnin et al., 2006), we concluded that a crossover design during this period would not disrupt our evaluation.

Patients were instructed to weigh 10 mL of gargle solution using a measuring cup and hold the solution in their mouth for 1 min and spit it out. Patients should avoid drinking water or rinsing their mouths for at least 5 min after using the gargle. To comply with the study protocol, patients were advised to wait at least 15 min between administrations. The daily dose was limited to one bottle (100 mL), and patients were advised to gargle with ibuprofen before every meal.

### 2.7 Concomitant medications

From POD 1, a single 60 mg tablet of loxoprofen sodium was administered for pain as required, with a maximum daily dosage of 180 mg. To minimize potential confounding effects of loxoprofen, a minimum interval of 6 h was maintained between the administration of loxoprofen sodium tablet and ibuprofen or placebo gargle on PODs 1–5.

### 2.8 Random allocation

The allocation of patients to the IP or PI group was conducted in a 1:1 ratio using the permutation random block method, with stratification based on the need for additional maxillary third molar extraction alongside mandibular third molar extraction. The block size was kept confidential to maintain blinding. The allocation sequence for the randomization method was generated by the biostatistician (T. I.). Patients, care providers, outcome assessors, and biostatisticians were blinded. Randomization was performed on all eligible patients who provided consent, met the inclusion criteria, and did not meet any exclusion criteria.

### 2.9 Endpoints

The primary endpoint was the within‐subject difference in terms of the visual analog scale (VAS) score (ranging from 0 [no pain] to 100 [worst pain]). The scores were obtained immediately before and 5 min after the first use of ibuprofen or placebo gargle on PODs 1 and 2 (ΔVAS_5_ibuprofen_ − ΔVAS_5_placebo_).

The secondary endpoints were as follows:

1. Within‐subject VAS score before and 15 min after the first use of ibuprofen or placebo gargle on PODs 1 and 2 (ΔVAS_15_ibuprofen_ − ΔVAS_15_placebo_).
2. ΔVAS_5_ and ΔVAS_15_ of the first ibuprofen gargle on PODs 3–5.
3. The number of times of gargle use and analgesic drug (loxoprofen sodium) administration per day on PODs 1–5.
4. Safety assessment of ibuprofen gargle.

### 2.10 Assessment

According to a previous study [34], all patients were required to use a diary to record pain intensity using the VAS scores immediately before and 5 and 15 min after the first use of ibuprofen or placebo gargle. Moreover, they recorded the number of times of gargle use daily and any other treatment‐related adverse events (TRAEs). Safety was assessed based on data from the patients' diaries and physician's consultation records. The safety assessment period was extended from the start of gargle use until PODs 6–10 after discontinuation, following the Common Terminology Criteria for Adverse Events version 5.0 (https://ctep.cancer.gov/protocoldevelopment/electronic_applications/ctc.htm#ctc_50). Furthermore, the study was periodically monitored to ensure the protection of participants' human rights and welfare, compliance with the protocol and relevant regulatory requirements under the Clinical Trials Act, and accurate data collection.

### 2.11 Statistical analysis

#### 2.11.1 Sample size calculation

The sample size was 40 patients, with 20 each allocated to the IP and PI groups, which was determined based on a previous study involving patients with oral mucositis due to chemotherapy or chemoradiotherapy (Ioroi et al., 2020). The mean ΔVAS ± standard deviation (SD) of pain relief after 3 days of ibuprofen gargling was −12.8 ± 8.4 mm (*n* = 7). In the subgroup with a baseline VAS score of ≥30 mm, the ΔVAS ± SD after ibuprofen gargle was −15.6 ± 8.1 mm (n = 5).

In this study, the ΔVAS_5_ ± SD of ibuprofen and placebo gargles was −15.0 ± 12.0 and −7.0 ± 12.0 mm, respectively. Therefore, to obtain the within‐subject difference of −8.0 mm in ΔVAS_5_ (ΔVAS_5_ ibuprofen − ΔVAS_5_ placebo), SD of 12.0 mm, ratio of 0.8−1.2 for between‐ and within‐subject variance (Tango, 1981), alpha error of 0.05, and beta error of 0.1; 30 cases were required. Hence, with consideration of a possible withdrawal rate of 25%, 40 patients were enrolled.

#### 2.11.2 Analysis

The full analysis set included all randomized patients who received at least one dose of the study drug during PODs 1–5. It excluded those without baseline data or major protocol violations (e.g., absence of informed consent). If missing data were identified, the investigator contacted the patient to resolve missing values. If the within‐subject difference or ΔVAS could not be determined due to missing VAS scores, it was considered as 0. If data other than VAS scores were missing or incomplete, the analysis could not be performed.

Continuous data were tabulated and summarized using the mean, SD, and 95% confidence intervals (CIs). In addition, categorical data were summarized using counts and percentages. For primary analysis, within‐subject difference for treatment, carryover, and period effects was estimated based on ΔVAS_5_ on POD 1 and 2. For secondary analysis, within‐subject difference for treatment, carryover, and period effects were estimated based on ΔVAS_15_ on POD 1 and 2.

The full analysis set was used for the primary analysis of this study. Sub‐analyses were also performed to address any imbalances in patient background, considering the effect of covariates. All analyses were performed using R (R Core Team, 2022, version 4.2.0).

## 3 RESULTS

### 3.1 Characteristics of the patients

**Figure 1:**
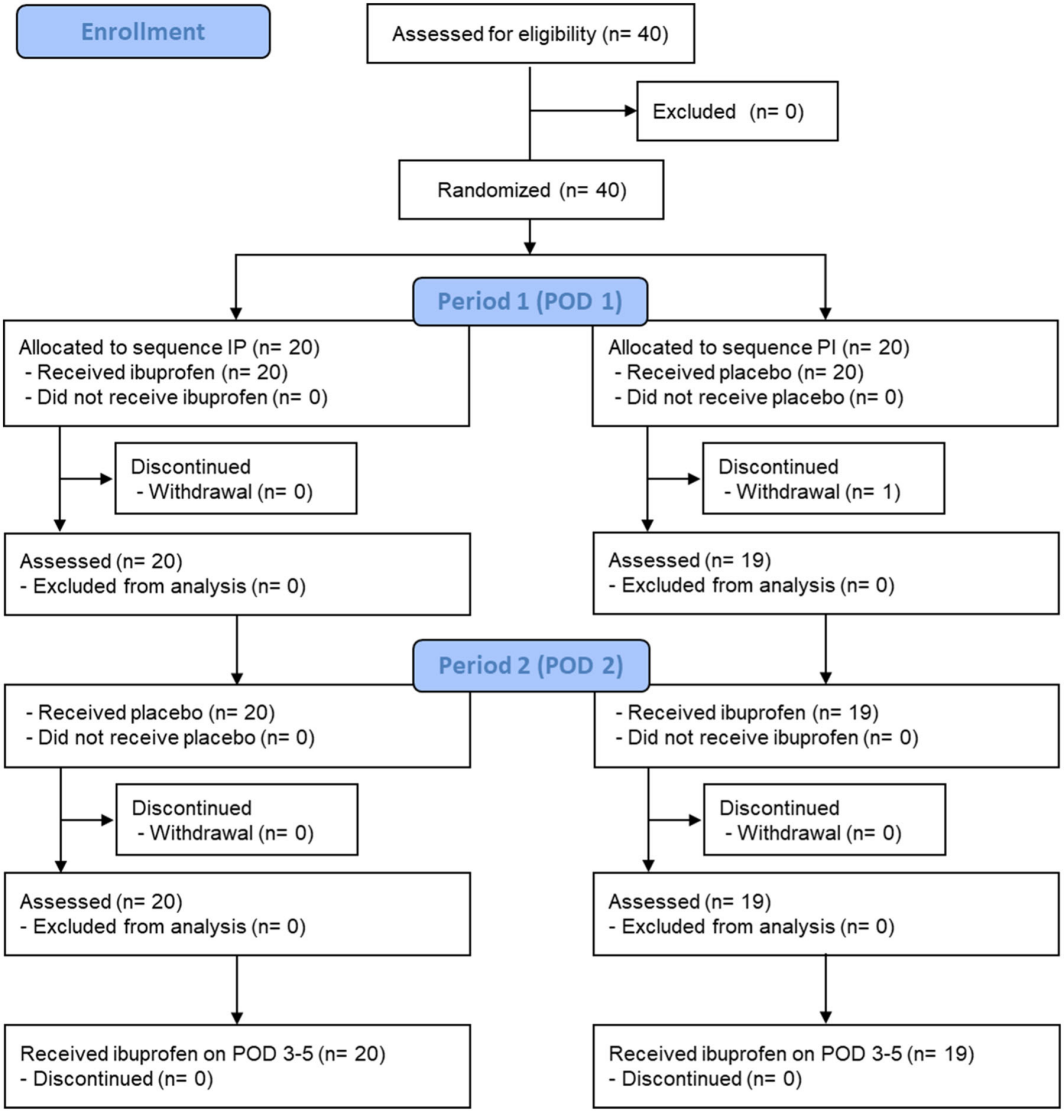
CONSORT flowchart. IP, ibuprofen–placebo group; PI, placebo–ibuprofen group.

Figure 1 depicts the flow diagram of the study. A total of 40 patients were enrolled between June 7, 2021, and May 26, 2022. The IP and PI groups comprised 20 patients each. However, one patient from the PI group withdrew their informed consent. The clinical characteristics of all 40 patients are presented in Table 1. Efficacy analysis was performed in 20 patients in the IP group and 19 patients in the PI group. Safety analysis was performed in 19 patients each in the IP and PI groups. None of the patients violated the study protocol to confirm periodic monitoring. The IP group included one patient who did not use gargle due to the absence of pain during the study period. The principal investigator did not conduct the unblinding procedure as no serious adverse events required emergency treatment.

**Table 1:** Patient Characteristics.

Characteristic	IP, *N* = 20^a^	PI, *N* = 20^a^
Age (year)	30.2 ± 9.7	28.1 ± 7.4
*Sex*		
Male	5 (25%)	9 (45%)
Female	15 (75%)	11 (55%)
*Reasons for extraction*		
Pericoronitis	14 (70%)	15 (75%)
Caries	2 (10%)	1 (5.0%)
Prophylactic	3 (15%)	3 (15%)
Other	1 (5.0%)	1 (5.0%)
*Extraction site*		
Mandibular	14 (70%)	14 (70%)
Both (mandibular and maxillary)	6 (30%)	6 (30%)
*Pell‐Gregory (space)*		
Class I	4 (20%)	1 (5.0%)
Class II	16 (80%)	19 (95%)
Class III	0 (0%)	0 (0%)
*Pell‐Gregory (depth)*		
Class A	15 (75%)	11 (55%)
Class B	5 (25%)	9 (45%)
Class C	0 (0%)	0 (0%)
*Winter*		
Mesioangular	3 (15%)	3 (15%)
Distoangular	1 (5.0%)	2 (10%)
Horizontal	6 (30%)	9 (45%)
Vertical	10 (50%)	6 (30%)
other	0 (0%)	0 (0%)
*Surgeon's experience (year)*		
3–5	2 (10%)	5 (25%)
6–10	18 (90%)	14 (70%)
≥11	0 (0%)	1 (5.0%)
Surgery duration (min)	27.3 ± 9.7	29.8 ± 11.6
Baseline VAS (mm)	42.3 ± 27.1	51.3 ± 23.8

Abbreviations: IP, ibuprofen to placebo; PI, placebo to ibuprofen; SD, standard deviation; VAS, visual analog scale.

^a^ Data are expressed as the number of patients (%) or Mean ± SD

### 3.2 Efficacy

Table 2 shows the primary endpoint. The within‐subject VAS_5_ scores of the IP and PI groups were 1.25 ± 12.0 and −5.26 ± 8.93 mm, respectively. The treatment effect of ibuprofen gargle was −2.01 mm (95% CI: −5.45 to 1.44, *p* = .246). The carryover and period effects were 9.84 (95% CI: −0.85 to 20.53, *p* = .070) and 3.26 (95% CI: −0.19 to 6.70, *p* = .063) mm, respectively.

**Table 2:** Results of the within‐subject difference (VAS5) and treatment effect.

	Treatment period		Within‐subject difference (I–P)
	ΔVAS_5 POD 1_	ΔVAS_5 POD 2_	
*I then P*			
Mean ± SD	−3.35 ± 5.58	−4.60 ± 10.16	1.25 ± 12.0
95% CI	−5.96 to −0.74	−9.36 to 0.16	−4.36 to 6.87
Sample size	20	20	20
*P then I*			
Mean ± SD	−6.26 ± 8.84	−11.53 ± 13.21	−5.26 ± 8.93
95% CI	−10.53 to −2.00	−17.90 to −5.16	−9.57 to −0.96
Sample size	19	19	19
*Treatment effect*			
Mean ± SD	‐	‐	−2.01 ± 10.62
95% CI			−5.45 to 1.44
Sample size	‐	‐	39
*t*‐test for paired samples	‐	‐	0.246

Abbreviation: CI, confidence interval; POD, postoperative days; SD, standard deviation; VAS, visual analog scale; ΔVAS_5 POD 1_, The changing VAS before and 5 min after the first use of the ibuprofen or placebo gargle on POD 1; ΔVAS_5 POD 2_, The changing VAS before and 5 min after the first use of the ibuprofen or placebo gargle on POD 2.

The within‐subject VAS_15_ scores of the IP and PI groups were −1.45 ± 18.31 and −7.16 ± 21.46 mm, respectively (Table 3). The treatment effect of ibuprofen gargle was −4.30 mm (95% CI: −10.77 to 2.16, *p* = .185). The carryover and period effects were 11.95 (95% CI: −2.13 to 26.01, *p* = .094) and 2.85 (95% CI: −3.61 to 9.32, *p* = .377) mm, respectively. In the sub‐analysis, we examined the impact of covariates on the treatment effect, including Pell–Gregory classification (space), Pell–Gregory classification (depth), Winter classification (particularly horizontal and vertical), surgeon's experience, and baseline VAS, which may contribute to the imbalances observed in Table 1. The adjusted treatment effect for each covariate was not statistically significant (Table 4).

**Table 3:** Results of the within‐subject difference (VAS_15_) and treatment effect.

	Treatment period		Within‐subject difference (I−P)
	ΔVAS_15 POD 1_	ΔVAS_15 POD 2_	
*I then P*			
Mean ± SD	−8.70 ± 15.04	−7.25 ± 13.09	−1.45 ± 18.31
95% CI	−15.74 to −1.66	−13.37 to −1.13	−10.02 to 7.12
Sample size	20	20	20
*P then I*			
Mean ± SD	−10.37 ± 15.19	−17.53 ± 15.49	−7.16 ± 21.46
95% CI	−17.69 to −3.05	−24.99 to −10.06	−17.50 to 3.19
Sample size	19	19	19
*Treatment effect*			
Mean ± SD	‐	‐	−4.30 ± 19.92
95% CI			−10.77 to 2.16
Sample size	‐	‐	39
*t*‐test for paired samples	‐	‐	0.185

Abbreviation: CI, confidence interval; POD, postoperative days; SD, standard deviation; VAS, visual analog scale; ΔVAS_15 POD 1_, The changing VAS before and 15 min after the first use of the ibuprofen or placebo gargle on POD 1; ΔVAS_15 POD 2_, The changing VAS before and 15 min after the first use of the ibuprofen or placebo gargle on POD 2.

**Table 4:** Subanalysis results of adjusted treatment effect by covariate.

Covariate	Adjusted treatment effect		*p* Value
	Estimate	95% CI	
Pell–Gregory (space)	2.07	−1.51 to 5.66	.249
Pell–Gregory (depth)	1.86	−1.69 to 5.40	.295
Winter (horizontal and vertical)	1.46	−2.83 to 5.74	.492
Surgeon's experience	2.38	−1.09 to 5.85	.173
Baseline VAS	1.71	−2.14 to 5.55	.373

Abbreviations: CI, confidence interval; VAS, visual analog scale.

**Figure 2:**
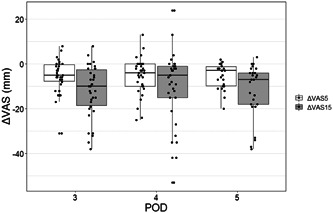
Distribution of ΔVAS_5_ and ΔVAS_15_ of the first ibuprofen gargle on PODs 3–5 according to dot chart and box plot. Abbreviation: POD, postoperative day; VAS, visual analog scale; ΔVAS_5_, change in VAS before and 5 min after the first use of ibuprofen or placebo gargle; ΔVAS_15_, change in VAS before and 15 min after the first use of ibuprofen or placebo gargle.

**Figure 3:**
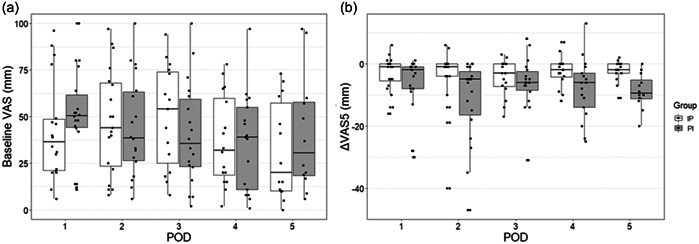
Distribution of baseline VAS (a) and ΔVAS_5_ (b) on PODs 1–5 according to dot chart and box plot. IP, ibuprofen–placebo group; PI, placebo–ibuprofen group; POD, postoperative day; VAS, visual analog scale; ΔVAS_5_, change in VAS before and 5 min after the first use of ibuprofen or placebo gargle.

Figure 2 shows the VAS_5_ and VAS_15_ of the first ibuprofen gargle on PODs 3−5. Figure 3 depicts the baseline VAS and ΔVAS_5_ on PODs 1−5. During all time points, ΔVAS was more likely to decrease; however, there were considerable variations among patients. The median numbers of daily gargle use on PODs 1−5 were as follows: POD 1: 2 (range: 0−5), POD 2: 2 (0−4), POD 3: 2 (0−4), POD 4: 1 (0−4), and POD 5: 1 (0−5). In addition, the median number of loxoprofen sodium tablets administered per day on PODs 1−5 was as follows: POD 1: 2 (range: 0−5), POD 2: 2 (0−4), POD 3: 2 (0−4), POD 4: 1 (0−4), and POD 5: 1 (0−5). There was no significant association between the ΔVAS scores and the frequency of gargle use.

### 3.3 Safety

The IP and PI groups did not present with serious adverse events or clinically significant complications (such as dry sockets) after extraction.

Seven adverse events were observed in five patients in the IP group. These adverse events were as follows: oral tingling sensation (*n* = 3), oral discomfort (*n* = 2), throat tingling sensation (*n* = 1), and tooth sensation (*n* = 1). In contrast, five adverse events were observed in five patients in the PI group. These adverse events were as follows: oral discomfort (*n* = 2), throat tingling sensation (*n* = 2), and oral tingling sensation (*n* = 1). During the placebo gargle use, oral discomfort (*n* = 3) and oral tingling sensation (*n* = 1) were noted.

All adverse events were grade 1 and were resolved at the end of treatment.

## 4 DISCUSSION

This single‐center, double‐blind, placebo‐controlled, crossover study investigated the efficacy and safety of ibuprofen gargle in reducing postoperative pain after mandibular third molar extraction. Our findings revealed that the primary endpoint of the ibuprofen gargle was not statistically significant, with a difference of −2.01 mm (95% CI: −5.45 to 1.44, *p* = .246) for VAS_5_ scores. Similarly, the VAS_15_ scores indicated a nonsignificant treatment effect of −4.30 mm (95% CI: −10.77 to 2.16, *p* = .185). Regarding safety, both the ibuprofen and placebo groups did not experience any severe adverse events or significant complications post‐extraction. This study aimed to evaluate the treatment effect without compromising the standard of care, and our findings provide valuable insights into the use of ibuprofen gargle in postoperative pain management.

The split‐mouth randomized controlled trial is a popular study design in oral health (Ceccheti et al., 2014; Christoffoli et al., 2021; Moranon et al., 2019). However, owing to contamination and carryover effect, treatment effects cannot be evaluated in this study design (Hujoel & DeRouen, 1992; Lesaffre et al., 2009). Based on our previous study (Ioroi et al., 2020), the median duration of pain relief was 20 (range: 10–210) min. Thus, we expected no period or carryover effects from the ibuprofen gargles and used a crossover design in this study.

Postoperative pain is a major complication of mandibular third molar extraction, necessitating symptomatic pharmacological treatment. Notably, oral administration of flurbiprofen, lornoxicam, and phytotherapeutic drugs has shown efficacy in managing pain following third molar surgery (Isola, Alibrandi, et al., 2019; Isola, Matarese, Ramaglia, Iorio‐Siciliano, et al., 2019). The first‐line treatment for pain and inflammation after third molar extraction is the continuous administration of NSAIDs as recommended (Bailey et al., 2013; P. A. Moore & Hersh, 2013). However, there are several side effects associated with the oral administration of NSAIDs (Gupta & Bah, 2016); therefore, topical NSAIDs may be preferable to minimize these side effects. Several studies have evaluated the effects of topical administration on postoperative pain. A small‐scale randomized study reported that the local injection of aspirin or acetaminophen into the mandibular third molar tooth socket reduced the pain area under the curve (0–8 h) compared with placebo (Moore et al., 1992). This was not the case with NSAIDs; however, a single‐blind randomized trial revealed that the group receiving topical ozone gel experienced significantly less postoperative pain than the control group (Sivalingam et al., 2017). We previously reported that ibuprofen gargles could be an effective local drug delivery system for pain relief after tooth extraction, with minimal systemic effects (Ioroi et al., 2020). However, further research is warranted to confirm this finding. Ibuprofen gargles may be considered as adjunctive therapy to manage pain before the next scheduled dose of NSAIDs following third molar extraction.

The primary endpoint of this study was not met because the results of ΔVAS varied widely among patients, which did not comply with our preliminary power analysis. This could be explained by the fact that the painful site after third molar extraction is deep in the tissues, unlike oral mucositis, and the local analgesic effect of the gargle was not as strong as expected. In addition, there is a potential for confounding effects from loxoprofen administration. Moreover, the Pell–Gregory classification, Winter's classification, surgeon's experience, and baseline VAS distributions were imbalanced across the two groups, which may affect the results. Therefore, future study designs aimed at improving the high SD of VAS scores may require a more limited study design or balanced patient background factors, such as Pell–Gregory and Winter classifications. Additionally, alternative methods to gargling should be considered.

To reduce the likelihood of systemic absorption, the ibuprofen gargle solution was held in the mouth for 1 min before being expelled. In addition, the concentration of ibuprofen used in the study (600 mg ibuprofen/100 mL) was comparable to the recommended maximum daily dose (600 mg) for oral ibuprofen administration. Thus, the concentration of any drug accidentally ingested could be less than the maximum daily allowance. This study did not detect apparent dry sockets due to delayed wound healing or renal impairment due to COX inhibition caused by ibuprofen gargling. The only TRAEs observed were unpleasant taste and irritation at the back of the mouth and throat. Ibuprofen gargle is considered acceptable from a safety perspective.

This study has some major limitations. First, we used VAS as our primary endpoint. Previous studies have employed objective outcomes such as the degree of swelling and opening, which should have been analyzed in this study (Isola, Matarese, Ramaglia, Cicciù, et al., 2019; Marques et al., 2014; Silva de Oliveira et al., 2016). Second, the sample size of this study was small. Phase II studies are frequently based on previous research. There have been no previous studies on the use of ibuprofen gargling after mandibular third molar extraction. Thus, when this study was planned, we could not estimate the primary endpoint and within‐subject VAS score before and 5 min after the first use of ibuprofen or placebo gargle on PODs 1 and 2. In addition, due to the nature of this study, there was a concern regarding the potential for confounding effects resulting from the administration of loxoprofen. Patients should ideally be evaluated in a completely analgesic‐free setting to assess the analgesic effects of ibuprofen gargle. However, this was not possible due to ethical reasons. Furthermore, patients with varying background factors were included in this study, and although we attempted to adjust for covariates by groups in the sub‐analysis, the small sample size may have limited our ability to draw meaningful conclusions. No common TRAEs associated with ibuprofen, such as gastrointestinal disorders, abnormal kidney function, or delayed wound healing, were observed in this study. Thus, it is possible that we overestimated the safety of ibuprofen gargle, and large‐scale studies are warranted to confirm this hypothesis. Based on the absence of unexpected or severe TRAEs, we believe that ibuprofen gargle therapy was well tolerated.

## 5 CONCLUSIONS

Despite its safe profile, ibuprofen gargle did not provide pain‐relieving effects when used after mandibular third molar extraction.

## AUTHOR CONTRIBUTIONS

*Conceptualization*: Takeshi Ioroi and Yasumasa Kakei. *Data curation*: Takeshi Ioroi. *Formal Analysis*: Takeshi Ioroi. *Funding acquisition*: Takeshi Ioroi. *Investigation*: Yasumasa Kakei, Tatsuya Shirai, Takahiro Ito. *Methodology*: Takeshi Ioroi, Yasumasa Kakei. *Project administration*: Takeshi Ioroi, Yasumasa Kakei, Takahiro Ito. *Resources*: Yutaro Okazaki. *Software*: Takeshi Ioroi. *Supervision*: Masaya Akashi Ikuko Yano. *Validation*: Yasumasa Kakei, Tatsuya Shirai. *Visualization*: Takeshi Ioroi, Takahiro Ito. *Writing—original draft*: Takeshi Ioroi, Yasumasa Kakei. *Writing—review and editing*: Takahiro Ito, Tatsuya Shirai, Yutaro Okazaki, Takumi Hasegawa, Masaya Akashi, Ikuko Yano. All authors have read and agreed to the published version of the manuscript.

## CONFLICT OF INTEREST STATEMENT

The authors declare no conflict of interest.

## ETHICS STATEMENT

The study was conducted according to the guidelines of the Declaration of Helsinki, and approved by the Clinical Research Ethics Committee of Kobe University (protocol code: C200024, and date of approval: March 23, 2021). Informed consent was obtained from all subjects involved in the study.

## Data Availability

The data that support the findings of this study are openly available in Supporting Information.
